# An extraoral approach to intraoral cooling–a feasibility study in non-cancer patients

**DOI:** 10.1186/s12903-023-03317-z

**Published:** 2023-09-08

**Authors:** L. Najaf, N. Borgvall, K. Vennman, J. Walladbegi

**Affiliations:** Department of Oral and Maxillofacial Surgery, Institute of Odontology, The Sahlgrenska Academy, University of Gothenburg, PO Box 450, 405 30 Göteborg, Sweden

**Keywords:** Chemotherapy, Cryotherapy, Oral mucositis, Stem cell transplantation

## Abstract

**Background:**

Cryotherapy, using ice chips (IC) is an effective strategy to prevent chemotherapy-induced oral mucositis (OM) in selected cancer patient cohorts. However, although effective, use of IC may cause adverse reactions, e.g., nausea, numbness, and shooting pain in the teeth, which could have an adverse impact on the medical treatment. Furthermore, IC requires water of good quality to minimize risk of serious systemic infections. To eliminate these disadvantages, novel cooling devices have emerged as alternative cooling modalities. Thus, the aim was to evaluate the efficacy and tolerability profile of extraoral cooling for intraoral temperature reduction.

**Subjects and Methods:**

This experimental pilot study was conducted at the Institute of Odontology, The Sahlgrenska Academy, University of Gothenburg, Gothenburg, Sweden. In total, six healthy volunteers were enrolled in this study. At baseline and following 30-, and 60 min of cooling with the extraoral cooling device, intraoral mucosal temperatures were measured using a thermographic camera, and a questionnaire related to tolerability was completed.

**Results:**

Following 30-, and 60 min of cooling, the intraoral temperature decreased with 2.0 °C and 2.3 °C, respectively. Extraoral cooling was well tolerated, and all subjects endured the entire cooling session of 60 min.

**Conclusion:**

Extraoral cooling reduces intraoral mucosal temperatures and is a well-tolerated cooling modality.

**Supplementary Information:**

The online version contains supplementary material available at 10.1186/s12903-023-03317-z.

## Introduction

The past decades have witnessed the emergence of a number of modalities for the management of oral mucositis (OM), a common debilitating adverse effect in conjunction with cancer therapy. Approximately 40% of all cancer patients receiving standard-dose chemotherapy (CMT), and up to 80% of those receiving high-dose CMT, as conditioning in preparation for hematopoietic stem cell transplantation (HSCT), develop OM [1, 2]. OM manifests itself clinically as erythema and/or ulceration and can lead to severe pain which may require intravenous opioid analgesics for pain relief [3, 4]. Furthermore, OM is associated with the need of total parenteral nutrition, prolonged hospitalization, impaired quality of life, and secondary infections, which may be potentially fatal [5, 6]. In aggregate, these may result in dose-reductions and could have an adverse impact on the medical treatment [7].

Despite the frequency of OM, its impact on patients, and the associated healthcare and economic burdens, there are currently few evidence-based interventions with confirmed efficacy. To date, mucositis management has largely been palliative, mainly aimed at reducing the symptoms of already established ulcers and preventing systemic complications. Although palliative strategies may be relevant once OM has become established clinically, the primary goal is prevention.

Current strategies for prevention of OM include the use of recombinant human Keratinocyte Growth Factor-1 (Palifermin) [8, 9], and low-level laser therapy (LLLT) [3]. Yet, cryotherapy (CT), using ice chips (IC) continues to be the most recommended modality for prevention of chemotherapy induced OM [6]. The proposed mechanism of protection is believed to be induction of local vasoconstriction, resulting in reduced blood flow and a lower tissue exposure to cytotoxic agents [10]. Other theories concerning CT also include that hypothermia protects the oral mucosa by lowering the metabolic activity of epithelial and basal cells, making them less susceptible to CMT [11]. However, despite favourable observations, use of IC as a preventive method in clinical practice is limited [12]. One explanation for this is that IC may entail adverse reactions such as chills, nausea, and shooting pain in the teeth, which could influence patient tolerability, leading to poorer adherence [13–15].

In addition, concerns have been raised that water to produce IC may contain microorganisms in concentrations which could increase risk of serious infections in immunocompromised cancer patients [16, 17].

To eliminate the disadvantages of IC, an intraoral cooling device (ICD) has been successfully developed. The ICD enables cooling at higher temperatures (6 to 22 °C; ± 2 °C) and has been proven equally effective as IC in terms of intraoral temperature reduction but with superior tolerability; This when set to operate at 8 °C [13]. Moreover, in a randomized controlled trial, the ICD (8 °C) as compared to IC, significantly reduced the grade of OM in lymphoma patients [18]. Nevertheless, despite its positive effects in clinical settings, the ICD comes with several limitations. These for example include swallowing difficulties, rubbing discomfort, and poor fit [19], likely attributed to intraoral anatomical differences which can complicate size optimization of the ICD.

Cooling, using an extraoral cooling device that comprises an anatomically fitted, temperature-controlled and water-circulating facemask has been used postoperatively in oral and maxillofacial surgery. In comparison to IC, it has shown reduction of post-operative facial pain, oedema, trismus as well as improved patient-reported satisfaction [20]. Thus, the aim of the current study was to evaluate the efficacy and tolerability profile of extraoral cooling for intraoral temperature reduction.

## Subjects and methods

### Study design

This study was an experimental pilot study to evaluate extraorally applied cooling for intraoral temperature reduction. All procedures were performed at the Institute of Odontology, The Sahlgrenska Academy, University of Gothenburg, Gothenburg, Sweden.

### Subjects

In total six (*n* = 6) dental students, three women and three men were recruited from the Institute of Odontology, The Sahlgrenska Academy, University of Gothenburg, Gothenburg, Sweden. Subjects were considered eligible to participate in the study if they met the following inclusion criteria: i) willing and able to provide written informed consent; ii) male or female, age ≥ 18 years; iii) had no medical diagnoses established by a physician; iv) did not consume any drugs with impact on the cardiovascular system; v) had no mucosal lesions which could affect the outcomes of the study. Exclusion criteria were: i) previous history of head and neck malignancy or other relevant pathologies; ii) previous history of radiation therapy to the head and neck; iii) use of tobacco or Swedish snuff.

### Tools and devices

#### Cooling device

**Fig. 1:**
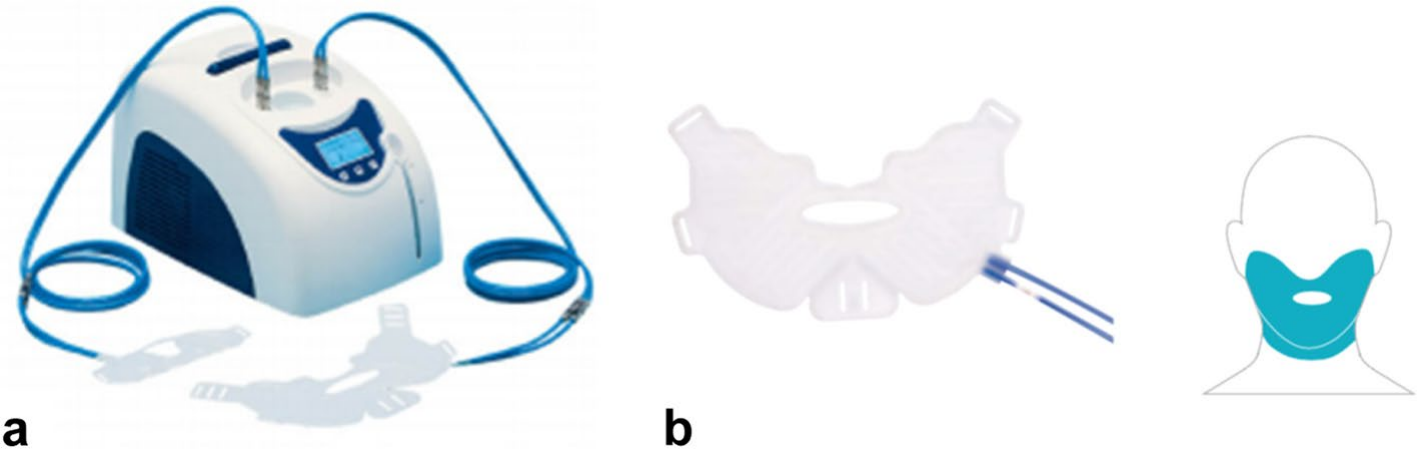
(**a**) Hilotherm Clinic® and (**b**) the disposable cuff (Lower Face Cuff with Link). Reprinted with permission from Hilotherm®

The extraoral cooling device (HILOTHERM Clinic®; Fig. 1a) was provided by Hilotherm® (GmbH, Argenbühl-Eisenharz, Germany). The disposable cuff was composed of an anatomically fitted, water-circulating facemask (Lower Face Cuff with Link; Fig. 1b). The cuff was made of soft plastic material with conduits through which water was delivered via a cooling and thermostat unit. Temperatures could be set between 10 – 35 °C. A water temperature, 10 °C (± 2 °C) was maintained throughout the entire study and any deviations from the default temperature were automatically adjusted by the system.

#### Questionnaire

The questionnaire (Additional file 1) consisted of six (*n* = 6) questions to assess the secondary endpoint of the study, i.e., tolerability of the cooling procedure. Questions included: the reason for not completing the cooling procedure; any adverse event; as well as space to share additional comments. Pain experienced during the cooling session was assessed with The Numerical Pain Rating Scale (NPRS; 0 indicating no pain; 10, indicating the worst imaginable pain). Prior to the study, to ensure face validity, all questions and response alternatives were tested and discussed with an independent group of participants (*n* = 5).

#### Tools and software for image analysis

**Fig. 2:**
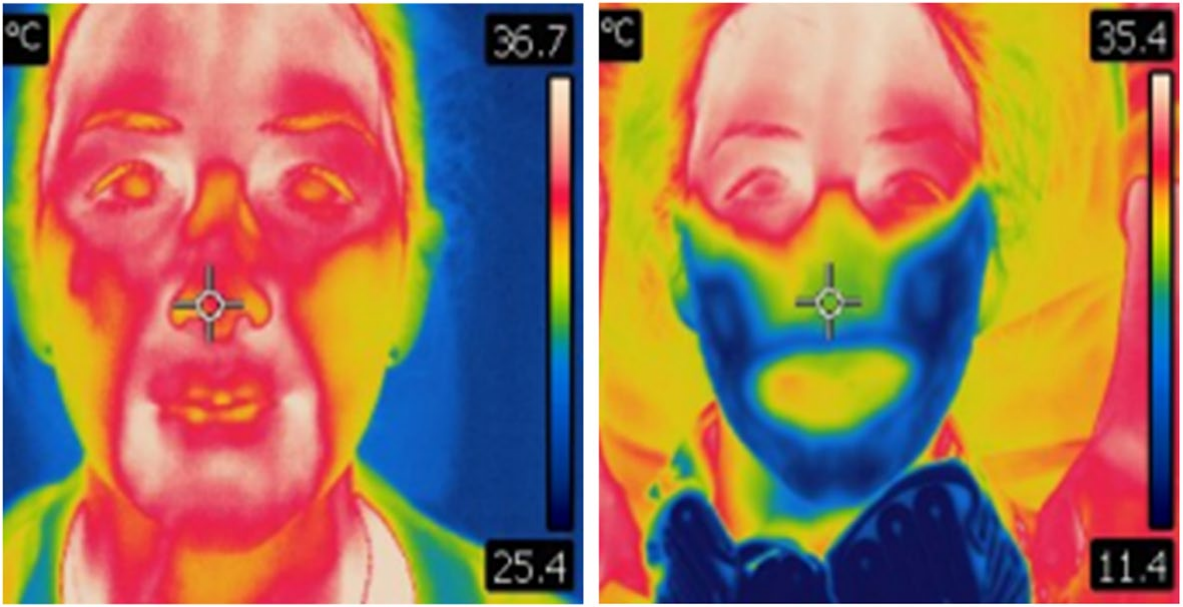
Thermographic images at baseline (left); and following 30 min of cooling (right) to illustrate the FLIR E60(bx) thermographic camera. Red colour indicates high temperatures and blue indicates low temperatures

The FLIR E60(bx) (FLIR Systems Inc., Wilsonville, OR, USA) is an accurate thermographic camera with a level of resolution (320 × 240 pixels) that allows detection of temperature differences corresponding to ≤ 0.05 °C (Fig. 2). The camera and its associated FLIR tools software were used to visualize and quantify temperature changes extraorally and in the intraoral mucosal regions of interest.

### Procedure and data collection

All measurements were performed in the same examination office (ambient temperature 22 °C ± 2 °C) at the Department of Oral and Maxillofacial Surgery, Institute of Odontology, The Sahlgrenska Academy, University of Gothenburg, Gothenburg, Sweden. Following inclusion, medical history was gathered, and all subjects underwent an intraoral examination. Subject characteristics including age, gender, weight, and height were registered, and BMI was calculated. Basic hemodynamics, including pulse, systolic and diastolic blood pressure were obtained non-invasively in the left upper arm with subjects sitting in an upright position, using the Omron M2 **(**Omron Healthcare Co. Muko, Kyoto. Japan). Subjects were then informed about the cooling procedure and hands-on demonstrations were set up to assure that subjects got acquainted with the cooling device. Upon cooling, the participants were asked to seat themselves in a dental chair in an upright or supine, 70° head-up position. Cooling continued for 60 min in one session. All participants were requested to refrain from eating and drinking at least 30 min prior to-, and throughout the cooling session. The cooling mask, set at 10 °C, was applied according to the manufacturer's instruction and proper skin contact was ensured by the investigators before cooling was initiated. At baseline and following 30-, and 60 min of cooling, temperatures were measured using the FLIR E60(bx) thermographic camera, in eight intraoral locations (right buccal mucosa; left buccal mucosa; upper labial mucosa; lower labial mucosa; dorsal tongue; base of the tongue; ventral tongue; and hard palate). In addition, to investigate whether there was any difference between extra-, and intraoral temperature reduction with the extraoral cooling device, extraoral images (frontal view) were captured at baseline, and at the same follow-up time points. Following image acquisition, the participants were asked to complete a questionnaire related to tolerability of the cooling procedure. All data was then exported, computer stored and subsequently analysed off-line by a blinded observer. The calculations were performed using the IBM® SPSS® Statistics software package (IBM SPSS Statistics version 25, IBM, Armonk, NY). All data were presented descriptively.

### Endpoints

The primary endpoint was:

1. To assess the efficacy of extraoral cooling for intraoral temperature reduction (°C).

The secondary endpoints were:

- To assess the efficacy of extraoral cooling for intraoral temperature reduction in risk surfaces, i.e., non-keratinized areas.
- To assess subject-related tolerability: well-tolerated being defined as ≥ 50 minutes of cooling.

## Results

In total, 12 subjects were evaluated for study participation, of whom 9 (9/12; 75%) fulfilled the inclusion criteria. Subsequently, 3 out of the 9 (3/9; 33%) were excluded due to the use of Swedish snuff, and 6 subjects were carried forward for the statistical analysis. In total, six cooling sessions were completed during this study, and 199 thermographic images were captured. From these, 162 images (162/199; 81%) were of such good quality that the mucosal temperatures could be clearly analysed. Subject characteristics are presented in Table 1.

**Table 1:** Subject characteristics at baseline. Quantitative parameters are presented as mean ± SD. BMI—body mass index; bpm—beats per minute; SBP—systolic blood pressure; DBP—diastolic blood pressure

**Subject characteristics**				
Gender	[F:M]	3	:	3
Age	[years]	25	±	2
Weight	[kg]	70	±	8
Height	[m]	1.7	±	0.1
BMI	[kg/m^2^]	23	±	2
**Vital parameters**				
Pulse	[bpm]	60	±	5
SBP	[mmHg]	113	±	8
DBP	[mmHg]	76	±	9

**Fig. 3:**
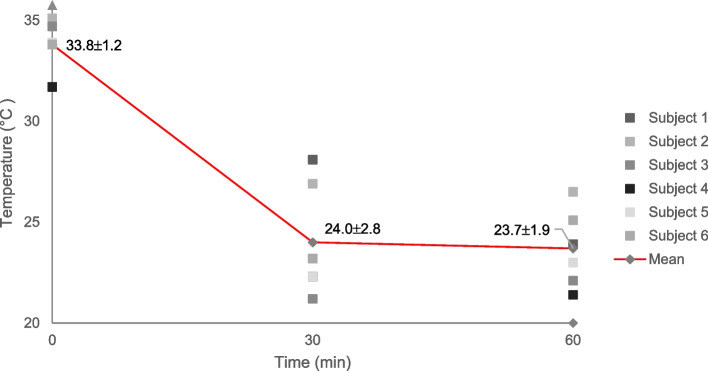
Extraoral temperature at baseline, and following 30-, and 60 min of cooling. Pooled subject data (red line) are presented as means and standard deviations

At baseline, prior to cooling, the mean extraoral temperature for all the subjects assessed was 33.8 ± 1.2 °C. Following 30-, and 60 min of cooling, a difference was demonstrated for each of the follow-up time points compared to baseline (24.0 ± 2.8 °C; 23.7 ± 1.9 °C), corresponding to a temperature reduction of 9.8 °C, and 10.1 °C, respectively (Fig. 3). An additional temperature reduction of 0.3 °C was observed between 30-, and 60 min of cooling.

**Fig. 4:**
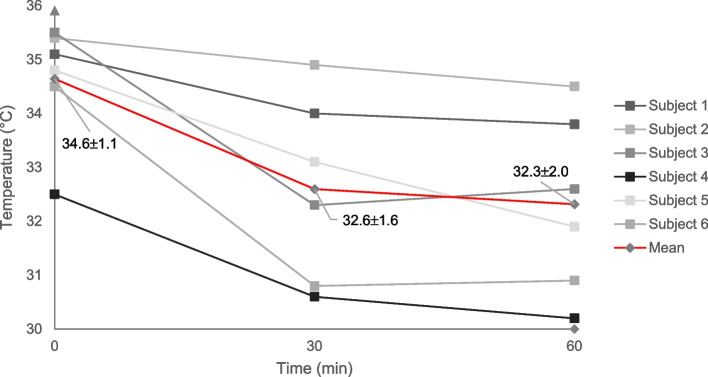
Intraoral temperature at baseline, and following 30-, and 60 min of cooling. Pooled subject data (red line) are presented as means and standard deviations

As for the primary endpoint, at baseline the mean intraoral temperature (eight locations) for all the subjects was 34.6 ± 1.1 °C. Following 30-, and 60 min of cooling, a temperature reduction was observed for both follow-up time points compared to baseline (32.6 ± 1.6 °C; 32.3 ± 2.0 °C), i.e., a temperature reduction corresponding to 2.0 °C, and 2.3 °C, respectively (Fig. 4). No difference was however observed between the temperature reduction at 30-, and 60 min of cooling. When evaluating each of the six subjects separately, the following temperatures were observed at baseline, 30-, and 60 min of cooling, respectively: subject 1 (°C; 35.1; 34.0; 33.8); subject 2 (°C; 35.4; 34.9; 34.5); subject 3 (°C; 35.5; 32.3; 32.6); subject 4 (°C; 32.5; 30.6; 30.2); subject 5 (°C; 34.8; 33.1; 31.9), and subject 6 (°C; 34.5; 30.8; 30.9).

Concomitant analysis of the risk surfaces, i.e., non-keratinized areas (right buccal mucosa; left buccal mucosa; upper labial mucosa; lower labial mucosa) presented a similar pattern, with regards to temperature reduction, i.e., with the greatest temperature reduction within the first 30 min.

**Fig. 5:**
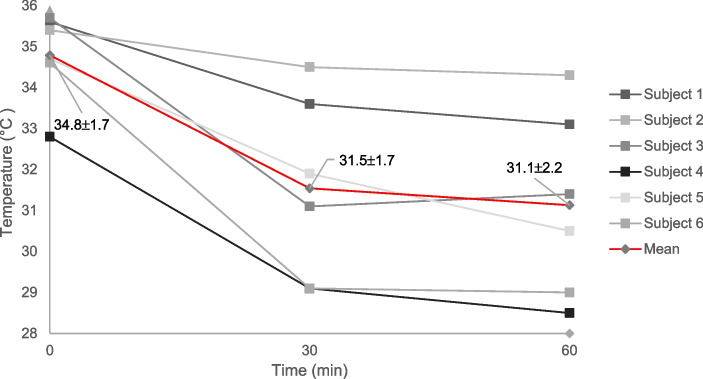
Intraoral temperature at baseline, and following 30-, and 60 min of cooling for risk surfaces, i.e., non-keratinized areas (right buccal; left buccal; upper labial; lower labial). Pooled subject data (red line) are presented as means and standard deviations

At baseline the mean intraoral temperature for the risk surfaces (four locations) for all subjects was 34.8 ± 1.7 °C. Following 30-, and 60 min of cooling, a temperature reduction was observed for both follow-up time points compared to baseline (31.5 ± 1.7 °C; 31.1 ± 2.2 °C, i.e., a temperature reduction corresponding to 3.3 °C and 3.7 °C, respectively (Fig. 5). No difference was observed between the temperature reduction at 30-, and 60 min of cooling.

The extraoral cooling device proved to be more effective regarding extraoral temperature reduction as compared to intraoral, demonstrating a difference following both 30-, and 60 min of cooling (7.8 °C).

All participants (*n* = 6) endured 60 min of cooling with the extraoral cooling device and the questionnaire related to tolerability was completed by all subjects. As for the subject-reported adverse events related to the cooling procedure, slight discomfort (*n* = 4), and coldness (*n* = 3), were the two most reported followed by poor fit (*n* = 2), numbness (*n* = 1) and difficulties speaking (*n* = 1). Two subjects (*n* = 2) reported ‘no discomfort’ as other comments. Four out of the six subjects did not experience any pain. One subject rated 1 on the NPRS and one subject reported 3.

## Discussion

The efficacy of cryotherapy (CT), using ice chips (IC) and intraoral cooling device (ICD) have been evaluated in a number of studies [13, 21]. However, although proven effective in prevention of CMT-induced oral mucositis (OM), both modalities present several limitations, mainly associated with patient-related comfort [18]. Therefore, we aimed to evaluate the efficacy of an extraoral cooling device, at first in healthy volunteers to assess its temperature reducing capacity, and based on the outcome in this study eventually in clinical trials in patients at risk of developing OM.

Until recently, IC has been the only available cryotherapeutic method for prevention of OM. Due to the nature of ice i.e., ice starts to melt when the temperature exceeds 0 °C, temperature controlled intraoral cooling has been difficult to implement. However, in a recent study [13] CT, using a novel ICD, which enables temperature regulation was evaluated. The possibility to regulate temperature has in turn raised further interest for research examining the exact level of intraoral temperature reduction needed for prevention of OM. As there is a probability that OM alleviation could be achieved at higher temperatures (8 °C) [18] than those obtained with intraoral cooling with IC, an extraoral approach would be more attractive due to its higher level of comfort.

This study showed that the extraoral cooling device reduced the intraoral mucosal temperature, after both 30- and 60 min of cooling when compared to baseline. Although not confirmed statistically, the main temperature reductions were seen in the buccal, and upper- and lower labial mucosae, respectively. These findings are promising since CMT-induced OM is mainly developed in the above specified risk surfaces (non-keratinized). This demonstrates that the extraoral cooling approach indeed can reduce the temperature of the oral mucosa. No differences were found between 30- and 60 min of cooling which suggests that merely 30 min of cooling is needed to reach a steady temperature.

It is noteworthy that the intraoral temperature reduction is superior when using the ICD as compared to extraoral cooling. This as the former resulted in a temperature reduction of 7.9 °C after 60 min of cooling [13], compared to 2.3 °C, and 3.7 °C in the present study for the intraoral mucosa as a whole and the non-keratinized areas, respectively. Although the differences in mean temperature reduction seems striking, in a recent in vivo study in rats [22] it was demonstrated that cooling to low temperatures (< 30 °C), which causes discomfort for the patient, may not be necessary to prevent OM. However, this is yet to be assessed in a clinical setting.

In the present study all six subjects endured the entire cooling session of 60 min with the extraoral cooling device indicating good tolerability. In comparison to already established strategies for OM prevention, most notably IC, but also the recently studied ICD, the subject-reported adverse events are likely less experienced when extraoral cooling is utilized. Fewer subjects experienced adverse events such as numbness and pain, which were the two most reported adverse events for IC in a previously conducted study [13]. Approximately 50 percent of the subjects experienced numbness with IC, compared to 17 percent of the subjects in this pilot study. In contrast, previous studies reviewed by the Cochrane Collaboration has reported few or no comfortability issues when using IC [21]. However, this could be explained by the fact that these studies focus on clinical outcomes, e.g., the degree and severity of OM, rather than patient reported outcomes such as tolerability of the cooling method.

As for the ICD difficulties in swallowing and rubbing discomfort were the two most common reported adverse events. Poor fit was also an issue associated with the ICD, which could potentially worsen OM due to rubbing damage to the intraoral mucosa. This in turn could lead to prolonged healing time and secondary infections. This issue is solved with the extraoral cooling device as it is applied externally and thus never in direct contact with the intraoral mucosa. The two most common adverse events reported with the extraoral cooling device were slight discomfort and coldness. None of the subjects experienced nausea, teeth sensations, headache, or vomiting sensation, which were also reported in the previous study on tolerability of ICs [13]. However, further studies are needed to establish a full tolerability profile due to the comparatively small sample size of this study.

In addition, as gagging is a common problem encountered during intraoral procedures, especially for younger patients [23], extraoral cooling has the main advantage over intraoral cooling that insertion of cooling device into the oral cavity is avoided. On the other hand, as compared to IC, the extraoral cooling device operates at higher temperatures which is likely to reduce the discomfort caused by IC cooling, leading to better compliance. Furthermore, there is a greater clinician/patient control enabled by the extraoral cooling device as compared to IC as there is no requirement for continual replenishment [20]. Ultimately, given the scarce effect of intraoral cooling for prevention of mucositis originating elsewhere along the gastrointestinal tract, our findings suggest that externally applied cooling could be of interest as a novel method for CT in areas otherwise difficult to access with conventional CT, e.g., the oro/hypopharynx.

This study has some limitations that should be acknowledged. First, even though the results in this pilot study seem promising, data are collected from a relatively small group of young and healthy participants. Thus, to draw firm conclusions further studies are needed in a larger population, preferably health compromised patients subjected to CMT.

Second, the facemask (Hilotherm) is available in one size, covering the lower parts of the face and above the upper lip. Individual differences in face shape, facial hair and amount of fat isolating the oral cavity may complicate a favourable fit and thus constitutes a potential source of measurement error. However, the degree to which this have affected the results is unknown. In addition, as the facemask is not individually fitted and is currently only available in one size, we believe there is room for further enhancement of the facemask, thus improvement in the potential temperature reduction. Additional studies would be beneficial to identify an optimal fit and approach for extraoral CT.

Third, the FLIR E60(bx) thermographic camera is not specifically designed for examining intraoral temperatures. It is noteworthy that approximately two minutes were used for the intraoral photo-series. During this time the facemask had to be removed and temperature recovery starts immediately after its removal. In the first 3 min, the temperature recovers by up to 50% [24]. Therefore, assumption can be made that the actual temperature reduction is somewhat larger than the data presented in this study. This can probably also explain the discrepancy seen in this study between extraoral and intraoral temperature reduction.

## Conclusion

Extraoral cooling reduces intraoral mucosal temperatures and is a well-tolerated cooling modality. However, prior to clinical use validation of extraoral cooling is required in larger cohorts.

### Supplementary Information

**Additional file 1.** Questionnaire to assess tolerability of the cooling procedure.

## Data Availability

The datasets used and/or analyzed during the current study are available from the corresponding author on reasonable request.

## Abbreviations

CMT: Chemotherapy
CT: Cryotherapy
HSCT: Hematopoietic stem cell transplantation
IC: Ice chips
ICD: Intraoral cooling device
LLLT: Low level laser therapy
NPRS: Numerical Pain Rating Scale
OM: Oral mucositis
