# Supplementary material for: An extraoral approach to intraoral cooling–a feasibility study in non-cancer patients
Source: BMC Oral Health. 2023 Sep 8;23:654. doi: 10.1186/s12903-023-03317-z (PMC10492390; doi:10.1186/s12903-023-03317-z)
Supplement: Supplementary file 1 — Additional file 1. Questionnaire to assess tolerability of the cooling procedure. [file 12903_2023_3317_MOESM1_ESM.docx]

**An extraoral approach to intraoral cooling – A pilot study**

1. Did you manage to keep the cooling facemask on for at least 50 minutes?

Yes (skip to question 4)

No

2. Approximately for how long did you keep the cooling facemask on?

1-20 minutes

21-30 minutes

31-40 minutes

41-49 minutes

50-59 minutes

3. Which of the below alternatives was the reason for not completing the cooling procedure? Tick the box/boxes that applies.

A  Coldness

B  Numbness

C  Headache

D  Pain

*Rate the experienced pain by circling the appropriate number.*

*No pain Worst pain imaginable*

*0 1 2 3 4 5 6 7 8 9 10*

E  Poor fit

F  Other…………………………….

4. Was it uncomfortable keeping the cooling facemask on?

No, not at all (skip to question 6)

No, not very much

Yes, a little

Yes, very much so

5. Did you experience any discomfort (several alternatives may be chosen)

A  Coldness

B  Numbness

C  Headache

D  Pain

*Rate the experienced pain by circling the appropriate number.*

*No pain Worst pain imaginable*

*0 1 2 3 4 5 6 7 8 9 10*

E  Poor fit

F  Other…………………………….
